# Blood pressure reactivity to mental stress is related to daily blood pressure variability in postmenopausal hypertensive women

**DOI:** 10.61622/rbgo/2025rbgo69

**Published:** 2025-10-21

**Authors:** Ana Clara Ribeiro Cunha, Caroline Pereira Garcês, Tássia Magnabosco Sisconeto, Juliene Gonçalves Costa, Mateus de Lima Rodrigues, Igor Moraes Mariano, Ana Luiza Amaral, Guilherme Morais Puga

**Affiliations:** 1 Universidade Federal de Uberlândia Faculdade de Educação Física Uberlândia MG Brazil Faculdade de Educação Física, Universidade Federal de Uberlândia, Uberlândia, MG, Brazil.; 2 University of Western Australia School of Human Sciences Perth Australia School of Human Sciences, University of Western Australia, Perth, Australia.; 3 Universidade Federal de Uberlândia Faculdade de Engenharia Elétrica Uberlândia MG Brazil Faculdade de Engenharia Elétrica, Universidade Federal de Uberlândia, Uberlândia, MG, Brazil.

**Keywords:** Hypertension, Climacteric, Postmenopause, Stroop test, Blood pressure, Blood pressure monitoring, ambulatory, Exercise test, Linear models

## Abstract

**Objective:**

This study aimed to investigate the relationship between blood pressure (BP) responses during a mental stress test with 24-hour ambulatory BP (ABPM) and its variability (BPV) in postmenopausal hypertensive women.

**Methods:**

BP reactivity under mental stress and ABPM were evaluated in 75 postmenopausal hypertensive women between 50 and 70 years, on non-consecutive days. We used the values of the variation (∆) of systolic BP (SBP) and diastolic BP from the reactivity test as independent variables, and the BPV indexes Average Real Variability (ARV), 24-hour SD, and SDdn (standard deviation weighted by daytime and nighttime duration) as dependent variables. In the multiple linear regression analysis, three models were tested: 1) without adjustment; 2) adjusted for time since menopause; and 3) adjusted for both nocturnal BP dipping classification and time since menopause.

**Results:**

The ARV index showed a significant association with ∆SBP variation in all models (model 1,2 and 3: β coefficient: 0.04, CI 95%: [0.00;0.08]).

**Conclusion:**

In conclusion, blood pressure variation during the mental stress test is related to ambulatory blood pressure variation, specifically to ARV in postmenopausal hypertensive women. These findings suggest that systolic blood pressure responses to mental stress may be a relevant predictor of daily blood pressure variability.

## Introduction

After menopause, there is an increase in risk factors for cardiovascular and metabolic diseases, such as elevated blood pressure (BP).^(1)^ This rise is attributed to the reduced cardioprotective effect of estrogen, increased oxidative stress, excessive visceral body fat, and a more sedentary lifestyle during this stage of a woman's life.^(2)^ Consequently, the incidence of arterial hypertension also increases postmenopause.^(3)^ This condition is directly linked to functional and structural changes in target organs.^(4)^ Furthermore, it stands as the main modifiable risk factor for cardiovascular and chronic kidney disease, as well as premature death.^(4)^

In this context, ambulatory BP monitoring (ABPM) is an effective tool for diagnosing arterial hypertension.^(4)^ This method can reveal short and accentuated fluctuations in BP throughout the day, known as BP variability (BPV).^(5)^ Elevated BP fluctuations may be associated with the development of cardiac, renal, and vascular damage, as well as increased mortality.^(6)^ Studies demonstrate that high BPV, in particular from systolic BP (SBP), is correlated with arterial stiffness, once it is related to aging and increased SBP levels, regardless of diastolic BP responses.^(7)^

Moreover, the circadian BP pattern includes a characteristic reduction during sleep.^(8)^ This nighttime decrease is significant, where individuals showing a decline typically of 10% or more compared to their mean awake BP are categorized as "dippers".^(9)^ In contrast, those with less than a 10% decrease are classified as ‘non-dippers’, which is associated with target organ damage and is predictive of cardiovascular morbidity and mortality.^(10)^

Additionally, psychological stress during daily life also contributes to BPV.^(11)^ Increased reactivity and reduced recovery from mental stress may be linked to poor cardiovascular health, including hypertension.^(12^but results have been variable. This review aimed quantitatively to evaluate the association between cardiovascular responses to laboratory mental stress and subsequent cardiovascular risk status in prospective cohort studies. We searched general bibliographic databases, PsycINFO, Web of Science, and PubMed, up to December 2009. Two reviewers independently extracted data on study characteristics, quality, and estimates of associations. There were 169 associations (36 articles^)^ Furthermore, these responses not only raise immediate BP levels but also contribute to endothelial dysfunction, inflammation, and oxidative stress, all of which are detrimental to vascular health and contribute to the progression of cardiovascular disease.^(13)^

Therefore, understanding and managing stress-induced BP reactivity is crucial in preventing and treating hypertension and reducing the overall cardiovascular risk, especially in risk groups as postmenopausal women. Our aimed is to relate BP responses during a mental stress test with 24-hour ABPM variability in postmenopausal hypertensive women. Our hypothesis is that hypertensive postmenopausal women who present greater reactivity to mental stress tests have greater BPV.

## Methods

It is characterized as a cross-sectional study carried out from March 2018 to March 2020. The study include postmenopause women (amenorrhea of at least 12 months and follicle-stimulating hormone (FSH) > 40 mIU/mL), medicated hypertensive, aged between 50 and 70 years, and without the use of hormonal therapy. Recruitment was carried out through the dissemination of the project on social and traditional media (television and radio), and data collection took place at the Laboratory of Cardiorespiratory and Metabolic Physiology. Participants were excluded if they were using hormonal therapy, smoked, had chronic kidney disease, or had a history of stroke or acute myocardial infarction. The procedures were carried out at the *Faculdade de Educação Física of the Universidade Federal de Ub*erlândia. The assessments initially consisted of anamnesis, anthropometric and body composition measurements, and resting BP, measured on three non-consecutive days in the morning. The volunteers were submitted to BPV assessment under mental stress and ABPM on separate days.

Height was measured using a stadiometer (Sanny, São Bernardo do Campo, Brazil), and body mass, as well as its distribution, was measured using the InBody 230 bioimpedance analyzer (Seoul, South Korea) after an 8-hour fasting period. Waist circumference was measured above the umbilical scar with an inelastic flexible measuring tape (Filizola, São Paulo, Brazil) without tissue compression. The body mass index (BMI) was calculated through the value of body mass and height, using the formula: BMI = body mass (kg)/height^2^ (cm).

Resting BP was monitored using the OMRON® BP HEM-7113 automatic monitor (OmronHealthcare CO., Ltd., Kyoto, Japan). Three BP measurements were taken at each assessment moment and the average was considered for analysis.

The 24-hour BP assessment was performed using the Dyna Mapa+ Cardius® (Cardios Sistemas, São Paulo, SP, Brazil) device, configured to measure BP every 20 minutes during awake and sleep. The volunteers wrote down in a daily log the activities that could change BP that occurred while using the monitor. Awake and sleep values were determined individually, according to the periods recorded in each diary.

The SBP and DBP values of the ABPM were used in the assessment of the BPV. This way we will be able to compare the following variables between the groups:

- SDdn: BP standard deviation weighted by daytime and nighttime duration (SD = √Σ (BP_x_- BP_mean_)^2^ / n);
- SD 24h: standard deviation of the 24h BP;
- ARV: average real variability (AVR = Σ (Bp_x_-Bp_x-1_) / n) of BP during 24h;
- Percentage nocturnal dipping (BPdip=(BPawake mean - BPsleep mean) x 100 / BPawake mean). Individuals were classified as present dipping (dipper), attenuated or absent (non-dipper) when the pressure reduction between the periods of awake and sleep were ≥ 10%, < 10% and ≤ 0% respectively

The assessment of stress reactivity under mental stress was performed using the 3-minute Stroop protocol of words and colors,^(14)^ with reactivity calculated as the peak BP during the test minus the resting BP immediately before the test. During the test, the volunteers must speak the color of the letters present on the computer screen as quickly as possible. However, there is a divergence between the color of the letters, the background of the screen, and the formed word (the name of another color). At every 1-minute interval, BP was measured by the auscultatory method, with a stethoscope and a column of mercury.

Data has been presented as mean ± standard deviation and/or percentage. Multiple linear regression analyses were performed, considering the variation of SBP and DBP in the Stroop test as independent variables and the variables of ABPM and BPV as dependent variables. Three statistical models were developed: Model 1 without adjustments, Model 2 adjusted for postmenopausal duration, and Model 3 adjusted for both postmenopausal duration and dipper classification. The number of independent variables was chosen to respect the criterion of the "minimum number of individuals for analysis = 50+8*m", with "m" being the number of independent variables.^(15)^ The models were tested against multicollinearity of independent variables through the variance inflation factor test (VIF, with values no greater than 10) and through correlations between the independent variables. Strong correlations (r or rho above 0.75) were considered as evidence of multicollinearity. Thus, after the Shapiro-Wilk normality test, the variables were correlated using Pearson's or Spearman's correlation, in parametric and non-parametric variables, respectively. The adjustment levels of the models were compared based on the Akaike information criterion (AIC), with smaller values representing a better adjustment. The paired t-test was used to compare resting and peak BP values during the mental stress test. For variables that did not follow a normal distribution, the Wilcoxon test was applied for the same comparison. Stata 14.0 software was used and p<0.05 was adopted for the level of significance.

The study presented herein has been approved by the Human Research Ethics Committee of the *Universidade Federal de Uberlândia* #5.440.421 (*Certificado de Apresentação de Apreciação Ética*: 48046221.6.0000.5152), following the ethical principles for research involving human beings according to the Declaration of Helsinki.

## Results

Out of 160 eligible women, 40 didn't sign the consent term and 45 were excluded due to incomplete assessments, leaving 75 who completed all tests and were included in the analyses. Table 1 shows the general characteristics of the volunteers.

**Table 1:** General characteristics of volunteers

General characteristics		Mean ± SD or n(%)
	Age (years)	58.1±5.0
	Body mass (kg)	69.9±8.8
	Stature (m)	1.59±0.10
	BMI (kg/m²)	27.7±3.8
	Eutrophic	19(25)
	Overweight	36(48)
	Obesity class I	19(25)
	Obesity class II	1(1)
	Waist circumference (cm)	94.5±9.0
	Body fat (kg)	27.9±7.2
	Fat %	18.8±19.8
	Muscle mass (kg)	34.5±8.5
	Postmenopause time (years)	9.0±6.5
	Resting SBP (mmHg)	119±12
	Resting DBP (mmHg)	75±9
	Dipper	29(39)
	Non-dipper	46(61)
Antihypertensive drugs		
	Beta-blockers	17(23)
	ACE inhibitors	13(17)
	ARB	41(54)
	Diuretic	18(24)
	CCB	2(3)
	None	6(8)
Pathologies associated with hypertension		
	Hyperthyroidism	1(1)
	Hypothyroidism	7(9)
	Dyslipidemia	7(9)
	Diabetes Mellitus type 2	2(3)
	Depression and anxiety	5(7)

BMI: body mass index; SBP: systolic blood pressure; DBP: diastolic blood pressure; ACE inhibitors: angiotensin-converting enzyme inhibitors; ARB: angiotensin receptor blockers; BCC: calcium channel blockers

Table 2 presents the results of the ABPM measurements across 24-hour, awake, and sleep periods, along with the BPV indexes, and the BP values during the mental stress test, including rest, peak, and the difference among them (∆). There was a significant increase in SBP and DBP during the BP reactivity test (p < 0.05).

**Table 2:** Ambulatory Blood Pressure 24h: Awake and Sleep Phases, Variability, and Response to Stroop Mental Stress Test

ABPM (mmHg)		Mean ± DP
	24h SBP	119±10
	Awake SBP	121±10
	Sleep SBP	111±13
	24h DBP	72±8
	Awake DBP	74 ± 9
	Sleep DBP	65±10
BPV (mmHg)		
	ARV SBP	10.32±2.58
	SD 24h SBP	13.06±3.16
	SDdn SBP	12.13±2.74
	ARV DBP	7.50±1.75
	SD 24h DBP	10.13±2.52
	SDdn DBP	9.19±2.02
BP stress reactivity (mmHg)		
	Rest SBP	119±12
	Peak SBP	149±19*
	∆ SBP	30±15
	Rest DBP	75±9
	Peak DBP	98±18*
	∆ DBP	23±16

SBP: systolic blood pressure; DBP: diastolic blood pressure; ARV: average real variability of systolic blood pressure; SD 24: standard deviation of 24 hours value; SDdn: standard deviation day/night standard; ABPM: ambulatory blood pressure monitoring; Rest: initial measurement taken at rest; Peak: highest value among measurements; ∆: variation of measurements performed.

* Significant difference between resting and peak values during the mental stress test (p ≤ 0.05)

Table 3 shows the multiple linear regression analyses reveal that, in all models tested, ∆SBP significantly influencing the ARV index (Standardized β coefficient: 0.04, CI 95%: [0.00;0.08]).

**Table 3:** Models of multiple linear regression

∆ SBP						∆ SBP			
		R^2^	Coef.	95% CI	p-value	R^2^	Coef.	95% CI	p-value
Model 1	Awake	-0.013	-0.02	[-0.18;0.14]	0.79	-0.006	-0.04	[-0.17;0.08]	0.45
	Sleep	-0.009	-0.05	[-0.25;0.14]	0.57	0.001	-0.07	[-0.22;0.07]	0.31
	ARV	0.049	0.04*	[0.00;0.08]	0.03	-0.013	-0.00	[-0.02;0.02]	0.90
	SDdn	-0.010	0.01	[-0.03;0.05]	0.59	0.015	-0.02	[-0.05;0.00]	0.15
Model 2	Awake	-0.009	-0.03	[-0.20;0.13]	0.69	-0.007	-0.04	[-0.17;0.07]	0.45
	Sleep	0.039	-0.08	[-0.27;0.11]	0.40	-0.013	-0.07	[-0.22;0.07]	0.31
	ARV	0.037	0.04*	[0.00;0.08]	0.04	-0.27	-0.00	[-0.02;0.02]	0.91
	SDdn	-0.017	0.01	[-0.03;0.05]	0.65	0.002	-0.02	[-0.05;0.00]	0.15
Model 3	Awake	-0.009	-0.03	[-0.19; 0.13]	0.70	-0.015	-0.04	[-0.17;0.08]	0.51
	Sleep	0.416	-0.07	[-0.22; 0.07]	0.34	0.320	-0.02	[-0.15;0.09]	0.67
	ARV	0.034	0.04*	[0.00; 0.08]	0.04	-0.039	-0.00	[-0.02;0.02]	0.86
	SDdn	-0.031	0.01	[-0.03; 0.05]	0.66	-0.011	-0.02	[-0.05;0.00]	0.15

Model 1: no adjustment; Model 2: adjusted for postmenopausal time; Model 3: adjusted for postmenopausal time and dipper classification; Dependent variables: awake BP, sleep BP, ARV BP, SDdn; independent variables: ∆ SBP and ∆ DBP. Coef.: coefficient; CI: confidence interval; ∆SBP: systolic blood pressure variation under stress; ∆DBP: diastolic blood pressure variation under stress; ARV: average real variability; SDdn: standard deviation weighted by daytime and nighttime duration.

* p<0,05. All indices related to ∆ SBP were indices of SBP variability, and those related to ∆ DBP were indices of DBP variability

## Discussion

This study investigated whether there is a relationship between BP during the mental stress test and BPV in postmenopausal hypertensive women. Our findings suggest that greater SBP reactivity to mental stress may be related to higher 24-hour BP variability in hypertensive postmenopausal women measured by the ARV index, aligned with the predictions made in our hypothesis.

The ARV index can be influenced by stress factors and is considered an important tool for assessing BPV due to its sensitivity to short-term changes in BP.^(16)^ An increase in this SBP index is associated with a higher risk of cardiovascular diseases.^(17)^ Our result is in line with Schulte et al.,^(18)^ who found a positive correlation between baseline systolic BPV and SBP reactivity in both hypertensive and normotensive individuals. Hypertensive individuals, in particular, exhibited greater BPV compared to normotensive individuals, along with a more pronounced SBP reactivity to emotional stress. Farah et al.^(19)^ also reported, in mice, that acute stress was associated with short-term BPV. Additionally, Hjortskov et al.^(20)^ demonstrated that acute stressors in humans can lead to BP elevations that persist after a stressful event, which may contribute to an increase in BPV. Stressful situations are a part of everyday life for the general population, imposing an increased burden on the cardiovascular system.^(21)^ Studies suggest that an increased BP response to mental stress is associated with the development of hypertension and is also a predictor of carotid atherosclerosis in this population.^(22)^ It is important to highlight that during mental stress, hypertensive individuals have higher BP values compared to normotensive individuals, increasing the risk factors for the development of other cardiovascular diseases or complications of existing conditions.^(23)^

The relationship between BPV and mental stress, along with the underlying mechanisms, has been extensively studied. The study by Hamer and Steptoe demonstrated that exaggerated cortisol responses to mental stress are associated with an increased risk of developing hypertension in healthy individuals, as hyperactivity of the hypothalamic-pituitary-adrenal axis indicates that chronic stress plays a central role in BP regulation.^(24)^ Furthermore, hypertensive individuals exhibit impaired reactivity compared to normotensive individuals, indicating a reduced capacity for cerebral autoregulation during mental stress, which is associated with potential endothelial dysfunction.^(25)^ Therefore, postmenopausal hypertensive women are at greater risk for cardiovascular complications, as menopause is characterized by a decrease in estrogen release, which also reflects changes in endothelial function and BPV.^(26)^

Based on this, the association observed in our study may be mediated by greater activation of the sympathetic nervous system, which is known to increase BPV over 24 hours.^(27)^ Mental stress increases adrenaline secretion, generating greater stimulation for sympathetic nervous system activation in the heart, which may lead to increased BPV. These changes can trigger cardiac arrhythmias and myocardial infarction, acting as a trigger for sudden death in patients with heart disease.^(23,28)^ Additionally, other factors influencing the increase in BPV include aging, elevated BMI, and the redistribution of adipose tissue.^(29)^

In this sense, other health conditions may also be related to changes in BP, particularly other markers of cardiovascular risk. For example, almost half (48%) of the sample was overweight and 25% were obese. Hypertension and obesity are two of the most common diseases, often interrelated, and they can significantly impact cardiovascular health.^(30)^ Excess body weight typically raises BP, while weight loss usually results in its reduction. In addition to increasing the risk of hypertension, overweight and obesity contribute to cardiovascular risk through their effects on lipid levels, atrial fibrillation, heart failure, stroke, insulin resistance, and other cardiometabolic processes, as well as by increasing all-cause mortality.^(31)^ Furthermore, hemodynamic changes in obesity, along with abnormalities in lipid and glucose metabolism, appear to be linked to fat distribution rather than just body weight.^(32)^ Specifically, the risks associated with obesity-related abnormalities are greater in cases of abdominal obesity, and in our sample, the average waist circumference is 94.5 cm, above the 88 cm recommended by the World Health Organization.^(33)^

Another interesting fact observed in our sample, 61% were non-dippers, showing a high prevalence of this characteristic in this population. There was a positive association between poor sleep quality and the presence of hypertension, with mean SBP and DBP values being higher for individuals who slept poorly. Additionally, individuals with hypertension and non-dippers demonstrated significantly worse subjective sleep quality.^(34)^ Concomitantly, BP follows a circadian rhythm in which it reduces at night, and there is a higher incidence of stroke in non-dippers.^(35)^

These findings highlight the complex relationship between mental stress testing and BPV in postmenopausal women with hypertension. Nevertheless, this study has certain limitations. Being observational, it is not possible to entirely eliminate residual confounding, although the analyses were adjusted for major confounding factors to minimize this effect. Additionally, the absence of a normotensive group may interfere with the results. The cross-sectional nature of the study offers useful insights into associations and aids in generating hypotheses for future longitudinal research; however, it does not allow for causal inferences, making longitudinal studies or randomized trials necessary to confirm these results.

As clinical applicability, the findings of the present study suggest that BP responses to stressful events in hypertensive postmenopausal women may play a crucial role in BPV throughout the day, including nocturnal changes. In practice, the Stroop test can be an effective tool for assessing and monitoring systolic BPV under stress, providing a more rapid and practical alternative to 24-hour ABPM, as elevations in systolic values during the Stroop test may reflect similar increases in ABPM.

## Conclusion

In conclusion, BP variation during the mental stress test is related to ABPM variation, specifically to ARV in postmenopausal hypertensive women. These findings suggest that SBP responses to mental stress may be a relevant predictor of daily BPV.

## Data availability

: The authors did not make the data from this article available in repositories prior to submission.
